# Antimicrobial activity of RP-1 peptide conjugate with ferrocene group

**DOI:** 10.1371/journal.pone.0228740

**Published:** 2020-03-26

**Authors:** Natalia C. S. Costa, Julia P. Piccoli, Norival A. Santos-Filho, Leandro C. Clementino, Ana M. Fusco-Almeida, Sarah R. De Annunzio, Carla R. Fontana, Juliane B. M. Verga, Silas F. Eto, João M. Pizauro-Junior, Marcia A. S. Graminha, Eduardo M. Cilli

**Affiliations:** 1 Department of Biochemistry and Technological Chemistry, Institute of Chemistry, São Paulo State University (UNESP), Araraquara, Brazil; 2 Department of Clinical Analysis, School of Pharmaceutical Sciences, São Paulo State University (UNESP), Araraquara, Brazil; 3 Faculty of Agrarian and Veterinary Sciences, São Paulo State University (UNESP), Araraquara, Brazil; Harvard Medical School, UNITED STATES

## Abstract

Parasitic diseases are a neglected and serious problem, especially in underdeveloped countries. Among the major parasitic diseases, Leishmaniasis figures as an urgent challenge due to its high incidence and severity. At the same time, the indiscriminate use of antibiotics by the population is increasing together with resistance to medicines. To address this problem, new antibiotic-like molecules that directly kill or inhibit the growth of microorganisms are necessary, where antimicrobial peptides (AMPs) can be of great help. In this work, the ferrocene molecule, one active compound with low levels of in vivo toxicity, was coupled to the N-terminus of the RP1 peptide (derived from the human chemokine CXCL4), aiming to evaluate how this change modifies the structure, biological activity, and toxicity of the peptide. The peptide and the conjugate were synthesized using the solid phase peptide synthesis (SPPS). Circular dichroism assays in PBS showed that the RP1 peptide and its conjugate had a typical spectrum for disordered structures. The Fc-RP1 presented anti-amastigote activity against *Leishmania amazonensis* (IC_50_ = 0.25 μmol L^–1^). In comparison with amphotericin B, a second-line drug approved for leishmaniasis treatment, (IC_50_ = 0.63 μmol L^-1^), Fc-RP1 was more active and showed a 2.5-fold higher selectivity index. The RP1 peptide presented a MIC of 4.3 μmol L^-1^ against *S*. *agalactiae*, whilst Fc-RP1 was four times more active (MIC = 0.96 μmol L^-1^), indicating that ferrocene improved the antimicrobial activity against Gram-positive bacteria. The Fc-RP1 peptide also decreased the minimum inhibitory concentration (MIC) in the assays against *E*. *faecalis* (MIC = 7.9 μmol L^-1^), *E*. *coli* (MIC = 3.9 μmol L^-1^) and *S*. *aureus* (MIC = 3.9 μmol L^-1^). The cytotoxicity of the compounds was tested against HaCaT cells, and no significant activity at the highest concentration tested (500 μg. mL^-1^) was observed, showing the high potential of this new compound as a possible new drug. The coupling of ferrocene also increased the vesicle permeabilization of the peptide, showing a direct relation between high peptide concentration and high carboxyfluorescein release, which indicates the action mechanism by pore formation on the vesicles. Several studies have shown that ferrocene destabilizes cell membranes through lipid peroxidation, leading to cell lysis. It is noteworthy that the Fc-RP1 peptide synthesized here is a prototype of a bioconjugation strategy, but it still is a compound with great biological activity against neglected and fish diseases.

## Introduction

The world population has been increasing exponentially and might reach around 8.6 billion people by 2030 [1]. In developing countries, a fraction of the expanding population lives in areas with poor sanitation and infrastructure, which contributes to elevate the risk of contracting infectious diseases such as vector-borne diseases and food/ water-borne illness [2–5], among others. Leishmaniasis, a vector-borne disease caused by *Leishmania* spp., is endemic in 98 countries and its cutaneous form presents an incidence of 0.7 to 1.2 million new cases per year [6]. Despite the importance of this disease for the public health system, there are only a few antileishmanial drugs currently available, which are highly toxic and require long-term parenteral administration, leading to low patient compliance and a consequent increase in the number of circulating drug-resistant strains [7]. For bacterial infections, it is estimated that drug resistances might cause 300 million additional deaths and an extra cost of 100 billion dollars to the public health systems by 2050 [8], making the development of new drugs an urgent problem.

Several synthetic and natural products have been explored in order to discover new antileishmanial/antibacterial drugs [9–21]. In general, the process of drug discovery relies on the identification of bioactive compounds that target biochemical and metabolic pathways that are essential for pathogens viability and infectivity. However, this strategy might identify bioactive compounds with a short therapeutic use given the pathogens' capacity to adapt and evolve towards drug-resistant phenotypes. The changes in the pathogens induced by the therapy can include, among others, the modulation of gene content [22, 23], expression or mutation [19, 23, 24] and deletion of transporters genes [25, 26], chromosomal mutations or, more commonly for bacteria, the acquiring of antibiotic resistance genes from other bacteria via mobile plasmids or transposons [23, 27]. On the other hand, the apparent inability of bacteria to develop effective resistance mechanisms against antimicrobial peptides (AMPs) is associated with the AMPs main target, the biological membranes. AMPs usually interfere with pathogens survival by targeting their membrane, leading to membrane permeabilization and outflow of cellular contents, causing the death of the microbe. In general, natural or synthetic AMPs interact with negatively charged phospholipids present in the outer membrane of pathogens rather than the host cells, which presents neutral phospholipids (zwitterionic). Therefore, AMPs have been investigated regarding their antileishmanial and antibacterial properties [28–34].

Moreover, chemical modifications might be employed to improve potency, safety and stability of AMPs [35]. Herein, we explored the antileishmanial peptide RP1 and its conjugate Fc-RP1. This AMP is derived from the human chemokine CXCL4 and was previously characterized regarding its antileishmanial properties [36]. Thus, in order to improve the potency of the antileishmanial RP1, we coupled ferrocene at the N-terminus of the sequence. The ferrocene is a known anticancer, anti-*Trypanosome brucei*, anti-*Plasmodium falciparum* and anti-HIV molecule [37–39].

The development of new drugs through the synthesis of bioconjugates containing organic molecules such as ferrocene, has been increasing due to its biological potential. Ferrocene is a low cost organometallic molecule that shows low toxicity and reversible redox properties, its ability to modify the structure by replacing the aryl/heteroaryl nucleus with the ferrocene in an organic molecule confers a significant change in molecular properties such as solubility and hydro/lipophilicity, enabling biological action of bioconjugates derived from this compound [40].

Thus, it is important to assess how the bioconjugation strategy can be used as an alternative to potentialize the action of drugs against different pathogens, increasing their selectivity and decreasing their toxicity. The functionalization strategy of the ferrocene to the RP1 peptide can generate a ferrocenium radical cation, which can act on the plasma membrane of microorganisms, leading to its destabilization. This effect added to the formation of reactive oxygen species leads to oxidative damage to several cellular components [41, 42], which may optimize the cytotoxic effect of PAM on bacteria and Leishmania parasite, possibly due to the increase of the lipophilic nature of the peptide induced by the ferrocene, that consequently, increase the ability to penetrate the cell membrane. Therefore, all information suggests that the conjugation of the Ferrocene to the RP1 peptide is an interesting approach to increase the bioactivity of the antimicrobial peptide.

Here, the conjugate and RP1 peptide were evaluated regarding their structure and potency against *Leishmania amazonensis*, a causative agent of cutaneous leishmaniasis. We also evalueted the activity of both compounds against the pathogenic bacteria *Streptococcus agalactiae*, *Aeromonas hydrophila*, *Staphylococcus aureus*, *Enterococcus faecalis*, and *Escherichia coli*. Therefore, the work also aims to investigate whether the conjugated compound Fc-RP1 shows optimal activity against two different organisms related to major public health problems.

## Material and methods

### Synthesis of peptides

The peptide RP1 (ALYKKFKKKLLKSLKRLG-COOH) [36] was synthesized by solid phase peptide synthesis (SPPS) using Fmoc protocols on Wang resin [43]. The amino acid couplings were performed at threefold excess over the amino component in the resin, using diisopropylcarbodiimide (DIC) and hydroxybenzotriazole (HOBt). The Fmoc group was deprotected by 20% 4-methyl-piperidine/dimethylformamide (DMF). Ferrocene carboxylic acid, purchased from Sigma (purity 97%), was coupled to the N-terminus of the peptide by using HCTU activators (O- (6-chloro-benzotriazoyl-1-yl) N, N, N'-hexafluorophosphate) N'-tetramethyluronium hexafluorophosphate) and DIPEA (N, N, N-diisopropylethylamine). Cleavage of the resin and removal of the side chain protecting groups were performed with 95% TFA (trifluoroacetic acid), 2.5% H_2_O and 2.5% triisopropylsilane (TIS) for 2 h [44]. The peptide was precipitated with ether anhydrous, separated from soluble non-peptide material by centrifugation, washed 3 times with ether and extracted in a mixture of 0.045% (v/v) TFA/H_2_O and 0.036% (v/v) TFA /ACN (Acetonitrile). The obtained solution was lyophilized. The crude product was purified by HPLC system using a semi-preparative reverse phase Phenomenex C18 column (250 mm x 10 mm, 300 Å, particle size 5 μm). A linear gradient elution was employed from 10 to 40% of solvent B (0.036% (v/v) TFA/ACN) for 90 min (solvent A = 0.045% TFA/H_2_O). The flow rate was 5 mL min^-1^ at room temperature, and the injection volume was 5 mL, with the UV detection being carried out at 220 nm. The purity of the peptide was analyzed using an analytical Shimadzu system with reverse phase C18 Ultrasphere Phenomenex column (4.6 mm x 150 mm, 300 Å, 5 μm particle size), detection at 220 nm, using gradient method of 5 to 95% solvent B in 30 min with 1 mL min^-1^. The identity of the peptide was confirmed by ion-trap Mass Spectrometer using a Brucker system in positive mode.

### Circular dichroism

The peptides were structurally characterized at 60 μmol L^-1^ by circular dichroism (CD) spectra analyses in a range of 195–260 nm using a JASCO J-815 CD spectrophotometer on nitrogen flush in 1-mm path-length quartz cuvettes at 25°C. Circular dichroism experiments were also performed on LPC (lysophosphatidylcholine) at concentrations of 1 and 5 mmol L^-1^ or on POPC and POPC:POPS (9:1) at concentrations of 300 and 150 μmol L^-1^. The spectra were recorded as an average of five scans. The data were collected in millidegree and further converted to molar ellipticity [Ɵ] (in deg cm^2^ dmol^-1^) [45].

### Stability test

Peptides stability was evaluated through degradation assays in neutral (PBS-pH 7.4) and acidic (water containing 0.045% trifluoroacetic acid) solutions and incubated at 37°C for 0, 6, 8 and 24 h, then chromatographically analysed (HPLC- analytical Shimadzu system with reverse phase C18 Ultrasphere Phenomenex column (4.6 mm x 150 mm, 300 Å, 5 μm particle size), detection at 220 nm) (S1–S5 Figs).

### Antibacterial assays

The Gram-positive *S*. *agalactiae* (GenBank accession number: MH359095.1), *S*. *aureus* (ATCC 25923), *E*. *faecalis* (ATCC 29212) and the Gram-negative *A*. *hydrophila* (GenBank accession number: MH305534.1) and *E*. *coli* bacteria (ATCC 25922) strains, were used. The minimum inhibitory concentration (MIC) of the peptides was evaluated by broth microdilution technique, according to CLSI (Clinical and Laboratory Standers Institute) (CLSI, 2012). The peptides were serially diluted in Mueller Hinton Broth (Himedia, Mumbai, India) at concentrations ranging from 0.092 μmol L^-1^ to 69.38 μmol L^-1^ for RP1; 0.0842 μmol L^-1^ to 63.18 μmol L^-1^ for Fc-RP1 and 0.87 μmol L^-1^ to 652 μmol L^-1^ for Ferrocene in 96 wells plates (Kasvi—Curitiba, PR, Brazil) with a pre-established bacterium inoculum, and followed incubation for 24 h at 37°C, the absorbance was measured at 600 nm (Biotek® ELx800—Winooski, VT, USA). Three independent experiments were performed and the data were analyzed by applying one-way ANOVA with Tukey's post hoc test using GraphPad Prism Version 5.01 software (GraphPad Software Inc., La Jolla, CA, USA). The maximum coefficient of variation accepted was 25% and the confidence level was 95% (p < 0.05).

### Antileishmanial assays

Promastigotes of *L*. *amazonensis* (MPRO/BR/1972/m1841-LV-79) were kept at 28°C in liver-infusion tryptose (LIT) medium supplemented with 10% heat-inactivated fetal bovine serum, FBS (Gibco/Invitrogen) [16] [46,47]. For the bioactivity evaluation of the peptides against intracellular amastigotes, murine macrophages were first obtained from peritoneal cavity of Swiss mice, and the obtained cells were further infected with promastigotes of *L*. *amazonensis* at the stationary growth phase. The peritoneal macrophages were collected from Swiss mice previously inoculated intraperitoneally with 3 mL of 3% sodium thioglycolate. After three days, the animals were sacrificed in a CO_2_ chamber and the peritoneal macrophages extracted after injection of phosphate buffer, PBS (pH 7.2), into the peritoneal cavity, followed by slight massage and suction of its content using a syringe (5 mL). The collected cells were transferred to a sterile tube placed in ice. The cells suspension was centrifuged (500 xg) and the sediment, resuspended in RPMI 1640 medium (Gibco^®^) supplemented with 10% FBS, 25 mmol L^-1^ HEPES and 2 mmol L^-1^ L-glutamine, counted and seeded at a density of 5 x 10^5^ cells/well onto coverslips (13-mm diameter) previously arranged in a 24-well flat-bottom plates. The plates were kept for 6 h at 37°C in an atmosphere of 5% CO_2_ for cell adherence.

Adherent macrophages were infected with *L*. *amazonensis* at stationary growth phase at a proportion of 10 promastigotes: 1 macrophage and incubated for 8 h at 37°C in an atmosphere of 5% CO_2_. Non-internalized promastigotes were removed by PBS washing, when the infected culture was treated with different concentrations of peptides or the positive control amphotericin B (0.075 to 10 μmol L^-1^), dissolved in fresh complete RPMI medium and incubated, as above described, for 24 h. After incubation, the cells were methanol fixed and followed by Giemsa staining. The number of intracellular amastigotes was counted under a microscope. At least 200 macrophages were counted in each of the three slides and the determination of the ratio between infected and uninfected macrophages, enabling the determination of the IC_50_-AMA in relation to drug-free control [3]. At the end, the inhibitory concentration for 50% of the amastigote forms (IC_50_-AMA) in BioEstat software was determined by nonlinear regression, the data was expressed as mean ± SD of two independent replicates, and the obtained results converted to the infection index values, which were obtained as the ratio between the percentage of infected macrophages and the average number of amastigotes per macrophage.

### Cytotoxicity

Murine macrophages were incubated for 6 h in 96-well flat-bottom plates (TPP) for cell adhesion in complete RPMI. Peptides were diluted in complete RPMI and incubated for 24 h, 37 ºC, in 5% CO_2_-air mixture. The viability of the cells was then analyzed by the colorimetric MTT (3-4(4,5-dimethyl-2-thiazolyl)-2,5-diphenyl-2*H*-tetrazolium bromide) assay as previously described [15]. Because the method is based on the ability of living cells to reduce the MTT to MTT-formazan, which is spectrophotometrically read at 570 nm, the cells were prior washed in PBS in order to avoid any interaction of the compounds with the MTT. All the assays were carried out in triplicates and in two biological duplicates. Amphotericin B was the standard drug used as a positive control and the experiments involving animals were performed in concordance to protocol approved by the Institutional Ethics Committee - CEUA (Ethics Committee in the Use of Animals) protocol CEUA/FCF/CAr n° 03/2019.

The peptide concentration corresponding to 50% cells growth inhibition was expressed as CC_50_ in μM and determined by non-linear regression using Bioestat^®^ software. Additionally, the Selective Index (SI = CC_50macrophages_/IC_50leishmania_) was determined, indicating the selectivity of the sample to the parasite.

For assays with HaCaT (immortalized human keratinocytes, ATCC^®^PCS200-011^TM^) obtained from the cell bank of Rio de Janeiro (BCRJ), the cells were cultured in Dulbecco’s Modified Eagle Medium (DMEM), supplemented with 10% FBS and antibiotics (penicillin 100 UMl^-1^; streptomycin 0.1 mg mL^-1^). Cultures were maintained in an atmosphere of 5% CO_2_ at 37° C, until the cells reached the cellular density of (5x10^5^ cells mL^-1^) [48]. To adjust the concentration, the adhered cells were trypsinized, centrifuged at 1200 rp'm for 3 min, and then transferred to 96 wells plate (5x10^4^ cells per well), followed by 24 h incubation for complete cell adhesion. Afterwards, the culture medium was changed to a fresh one and the cells were treated with different concentrations of compounds: for RP1 varying from 231 μmol L^-1^ to 0.72 μmol L^-1^; Fc-RP1 210 μmol L^-1^ to 0.65 μmol L^-1^; and Fc 2174 μmol L^-1^ to 6.78 μmol L^-1^. The plates were further incubated for 24 h and the cytotoxicity performed by alarmable^®^ (Thermo Fisher Scientific) cell viability assay [49]. After treatment with the peptides for 24 h, the medium was discarded, and 100 μL of Alamar Blue dissolved in medium (10:1) was transferred to the 96-well microplate. The microplates were incubated at 37°C for 4 h, and absorbance was measured in a microplate reader (Epoch2, Biotek) in 570 nm (normalized to the 600 nm) [49]. Cell viability was determined based on the ratio of the absorbance between treated and untreated cells and the data were expressed as percentages [48, 50]. The experiments were made in triplicates.

### Hemolysis assays

Fresh red blood cells (RBC) from the Nile tilapia *Oreochromis niloticus* were collected, the plasma separated by centrifugation at 350 x g for 10 min at 28°C, and washed three times with PBS. Different concentration of the peptides was incubated with 4% (v/v) of RBC for two hours at 28°C under agitation (150 rpm) and the absorbance of supernatants was measured at 560 nm. As a positive control, a 1% Triton X-100 solution was used to obtain 100% hemolysis. The negative control was performed using 0.15 mol L^-1^ PBS pH 7.2. The selectivity index was calculated based on the ratio between HA/MIC [51].

### Vesicles preparation and permeabilization assay

Large unilamellar vesicles (LUVs) composed by POPC (1-palmitoyl-2-oleoyl-sn-glycero-3-phosphatidylcholine) or by POPC and POPS (1-palmitoyl-2-oleoyl-sn-glycero-3- serine) (9:1) were prepared according to the method described in Lorenzón, EN et al 2014.

The release rate of CF from vesicles was measured by the fluorescence intensity with wavelength set at 495 nm excitation and 517 nm emission after the addition of the peptides RP1 at 10 μmol L^-1^ and 50 μmol L^-1^, Fc-RP1 at 0.1 μmol L^-1^, 1 μmol L^-1^ and 10 μmol L^-1^ and ferrocene carboxylic acid at 0.7 mmol L^-1^. The data were acquired using a Fluorolog-3 FL3-122 spectrofluorometer equipment (Horiba Jobin Yvon) and all experiments were performed in duplicate at 25°C.

## Results

### Synthesis of peptides

The peptide RP1 [36] was synthesized by SPPS, purified by HPLC semi-preparative and characterized by HPLC analytical and mass spectrometry (S1 Fig).

In order to obtain the RP1 coupled to ferrocene, the bioinorganic compound was coupled to the N-terminus of the peptide using three-fold excess of ferrocene carboxylic acid and HCTU, and six- fold excess of DIPEA for 4 h. The Fc-RP1 conjugated derivative was successfully obtained as demonstrated in (S2 Fig). The presence of additional peaks in the chromatogram is attributed to the ferrocene degradation in acidic medium, as previously reported [52].

### Circular dichroism

**Fig 1:**
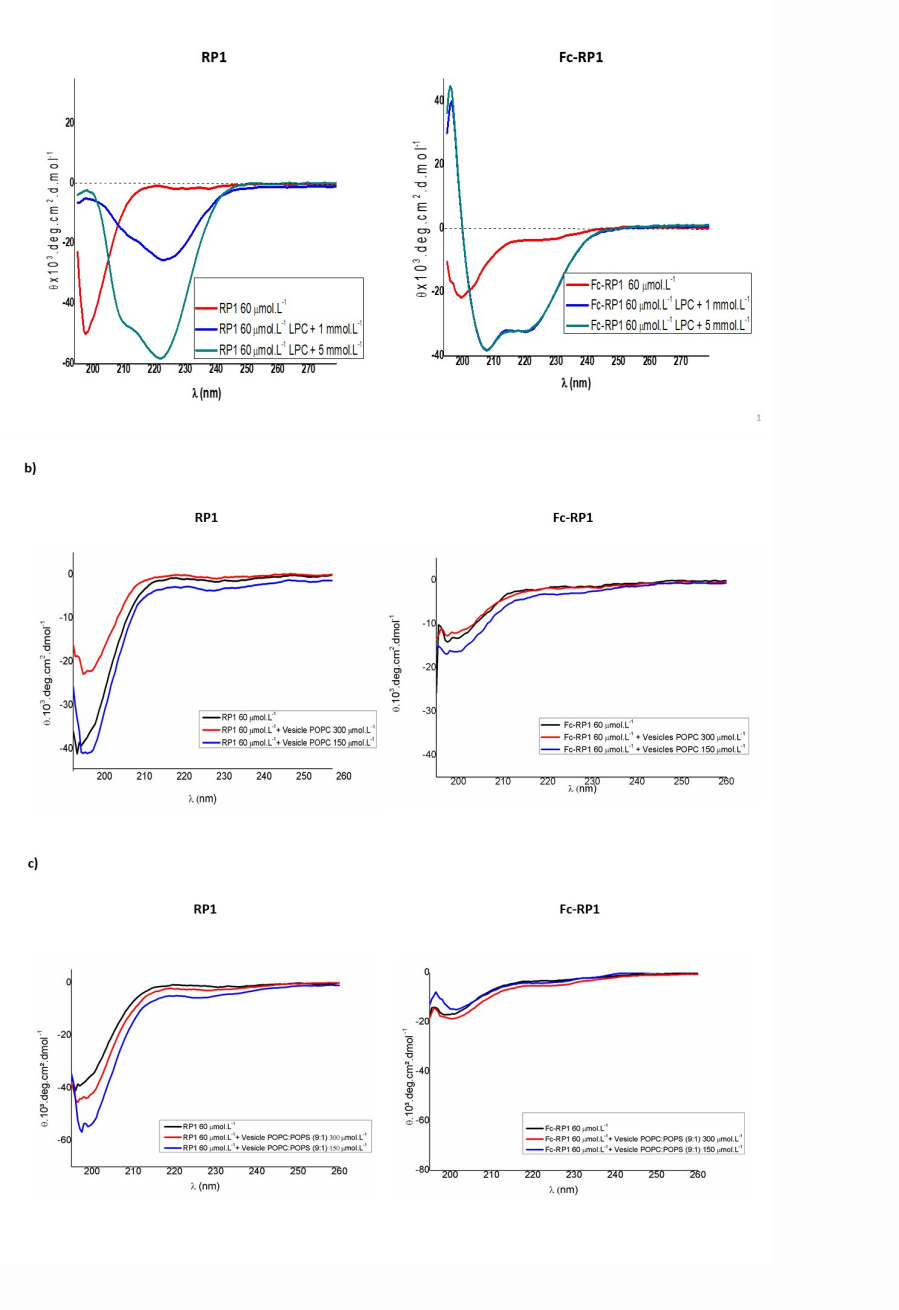
CD spectra of RP1 and Fc-RP1. (a) RP1 and Fc-RP1 in PBS solution and LPC at concentrations of 1 and 5 mmol L^-1^. (b) Peptides in POPC unilamellar vesicles at the concentrations of 300 and 150 μmol L^-1^ of lipids. (c) Peptides in POPC: POPS vesicles (9:1 / m:m) also at the concentrations of 300 and 150 μmol L^-1^ of lipids. The peptides were evaluated in concentration of 60 μmol L^-1^ dissolved in PBS.

For PBS solution, RP1 and Fc-RP1 peptides presented disordered structures (Fig 1A). In the presence of LPC, a membrane mimetic environment, in a concentration of 1 mmol L^-1^, which is below the critical micellar concentration (CMC) [53], RP1 presented a mixture of structures: α-helix, characterized by negative band at 222 nm; and β-sheet structures containing a small shoulder negative band around 215 nm [54, 55]. In 5 mmol L^-1^ of LPC, above the CMC, the peptide increased the content of α-helixes structures [56], showing well-characterized positive and negative bands at 190 nm and 208/222 nm, respectively. The CD spectra in micelles showed aggregation of RP1, considering a characteristic band at 222 nm higher than in 208 nm [56, 57]. The absence of a positive band in 190 nm suggests the presence of additional structures. The CD spectra analyses of Fc-RP1 showed some structural differences. The ferrocene promotes a decrease in the level of aggregation of the peptide and the presence of defined α-helical structures, as can be depicted by the presence of negative (208/222 nm) and positive (195 nm) bands. The obtained intensities of the peaks at 222 nm (θ_222_) and 208 nm (θ_208_) lead to a θ_222_/θ_208_ ratio less than one, suggesting absence of aggregation among the peptide chains [58, 59].

Micelles are not real mimetics of membranes. Thus, in order to discard any influence of the high curvature of these structures, the peptides were evaluated in unilamellar vesicles, mimicking both microorganisms/human eukaryotic (POPC–zwuitterionic lipid) or fungal/parasitic (POPC:POPS–negative charge) cell membranes [60, 61]. The outer mammalian membranes are composed of lipids that primarily have neutral charges (POPC). The model used for fungal bilayer included the zwitterionic phospholipid POPC, as a neutral charge, and POPS, which belongs to the most abundant anionic phospholipid group in the yeast cell membrane. PC and PS are phospholipids also found in high proportions in the cell membranes of other fungi [62]. In both vesicles, the peptides did not present a well-defined secondary structure (Fig 1B and 1C), which could be attributed to a smaller initial interaction between them, making the structural changes small.

### Antileishmanial potential of Fc-RP1

**Fig 2:**
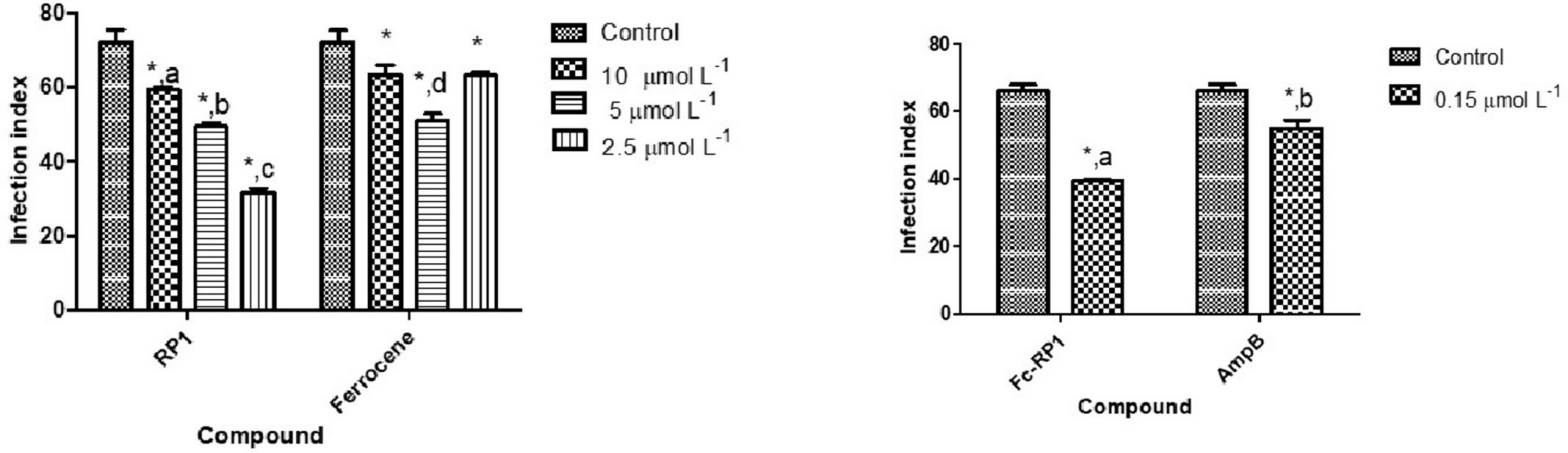
In vitro effect of a) RP1 and ferrocene carboxylic acid and b) Fc-RP1 and Amphotericin B against *L*. *amazonensis* intracellular amastigotes. The infection index was calculated after 24 h treatment at the IC_50_ of each compound. The negative control is *L*. *amazonensis* intracellular amastigotes not treated with the compounds. The data are expressed as averages plus standard deviations (SD) from two independent experiments (P <0.01).

In order to evaluate the antileishmanial activity of the derived RP1coupled to ferrocene, Fc-RP1 was tested against intracellular amastigotes of *L*. *amazonensis*. The results indicate that Fc-RP1 (IC_50_ = 0.25 μmol L –^1^) is more potent than both RP1 (IC_50_ = 1.25 μmol L^–1^) and ferrocene carboxylic acid (IC_50_ = 4.4 μmol L^–1^). The activity of the compounds on non-infected macrophages was also evaluated. The CC_50_ showed high values for all compounds as compared with IC_50_. In this experiment the Fc-RP1 conjugate was the more active one. Although the conjugate presents a higher activity against amastigotes, its selective index was 70, a small difference in relation to the peptide RP1 (SI = 80). In this case, the peptide was more selective to the amastigotes rather than Fc-RP1 (Fig 2 and Table 1). In comparison with positive control amphotericin B, the Fc-RP1 showed lower IC_50_ and higher SI, indicating the potential of this molecule for this type of application.

**Table 1:** Evaluation of the anti-amastigote activity in *Leishmania amazonensis* and cytotoxicity with mouse peritoneal macrophages Swiss.

Compounds	IC_50-AMA_ (μmoL L^-1^)*	CC_50_ (Peritoneal Macrophages) (μmoL L^-1^)*	IS
RP1	1.25±0.70	>100±0.02	80
Fc-RP1	0.25±0.38	17.3±0.03	69
Ferrocene carboxylic acid	4.4±0.91	>100 ±0.03	22
Amphotericin B	0.63±1.17	17.73±0.05	28

*Results are expressed by the mean of the duplicates plus the standard deviation.

### Antibacterial and cytotoxic potential of Fc-RP1

**Fig 3:**
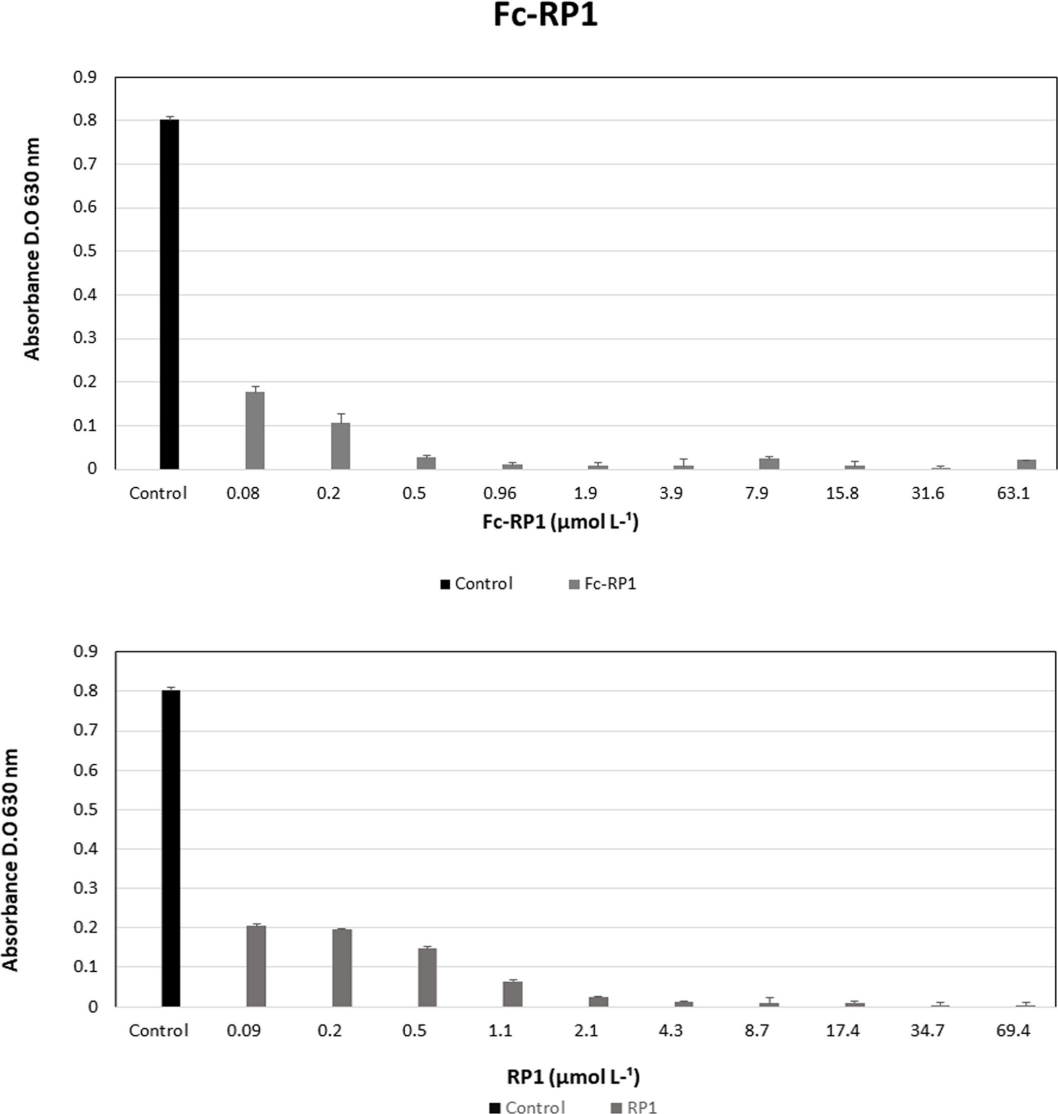
Antibacterial assays of the peptides RP1 and Fc-RP1 in S. *agalactiae* (Gram-positive). Peptides were diluted in ultrapure water and positive control (bacterium and Mueller Hinton broth). Data are expressed as averages plus standard deviations (SD) from three independent experiments (P <0.05) a statistically significant difference of the different experimental points. According to Tukey's test, the tested peptide concentrations differ statistically from each other, being represented by different letters on the tested drug concentrations.

RP1 and Fc-RP1 showed antibacterial activity against *S*. *agalactiae*, Gram-positive pathogenic bacteria from fish. Indeed, RP1 presented a MIC of 4.3 μmol L^-1^, whilst Fc-RP1 was four times more active (MIC = 0.96 μmol L^-1^), indicating that ferrocene improved the antimicrobial activity against the Gram-positive bacteria (Table 2 and Fig 3). However, the peptide and the conjugate does not show activity on *A*. *hydrophila* (Gram-negative bacteria) (S4 Fig).

**Table 2:** MIC (μmol L^-1^) values of RP1 peptide, FcRP1 and ferrocene.

	RP1	FcRP1	Fc
Strain	CIM	CIM	CIM
***E*. *faecalis***	8.7	7.9	> 652
***S*. *aureus***	69.4	3.9	> 652
***E*. *coli***	8.7	3.9	> 652

MIC: Minimum Inhibitory Concentration. Fc: ferrocene.

**Fig 4:**
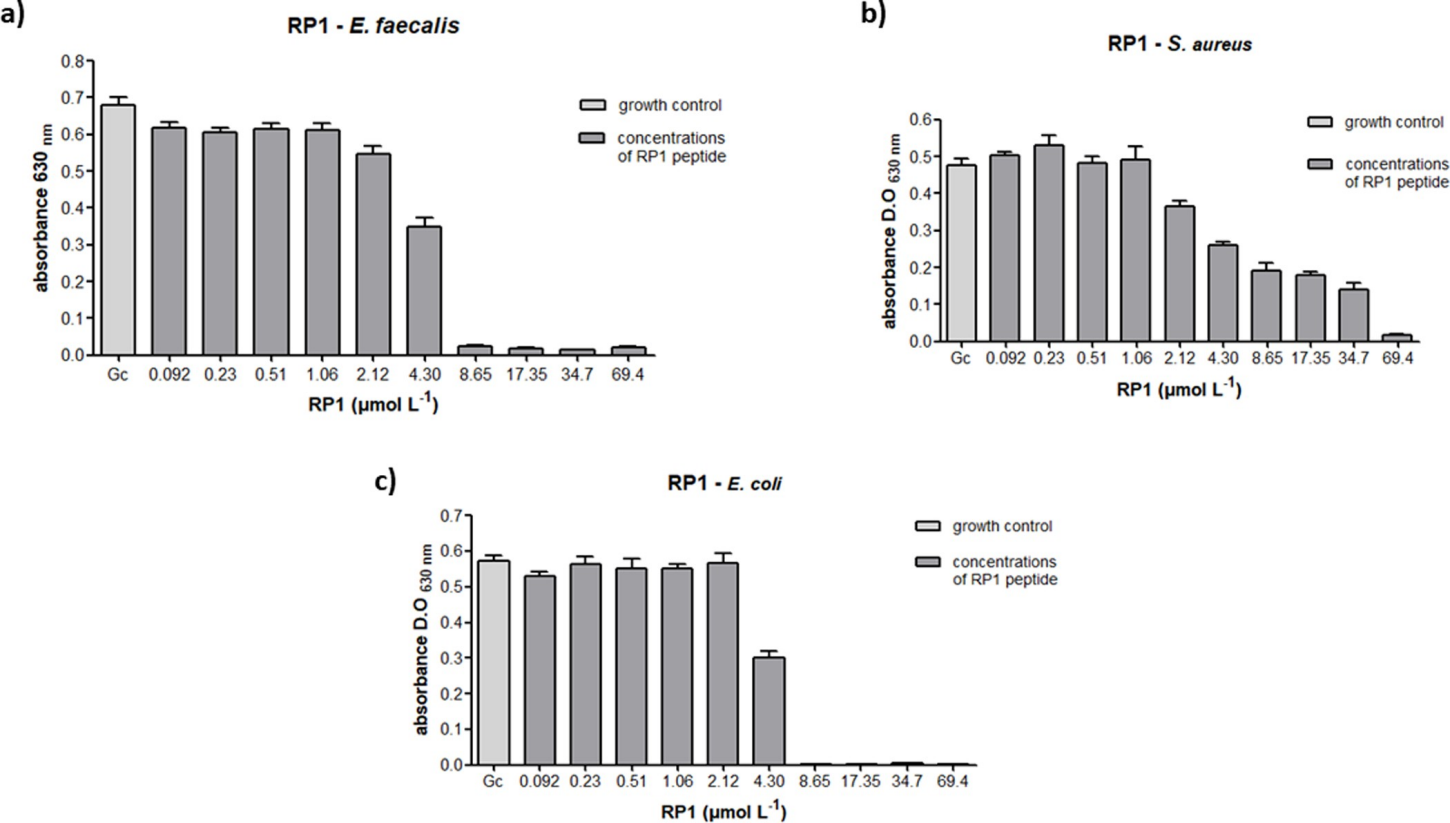
Antibacterial assays with peptide RP1 against (a) *E*. *faecalis* (b) *S*. *aureus* and (c) *E*. *coli*. The methodology followed the guidelines of the Clinical and Laboratory Standers Institute (CLSI, 2012). Three independent experiments were performed and the data were analyzed by applying the one-way ANOVA with Tukey's post hoc test using GraphPad Prism Version 5.01 software (GraphPad Software Inc., La Jolla, CA, USA). The maximum coefficient of variation accepted was 25%, the confidence level was 95% (p <0.05) and ***p<0.001, *p<0.05; **p<0.01; ns: not significant.

**Fig 5:**
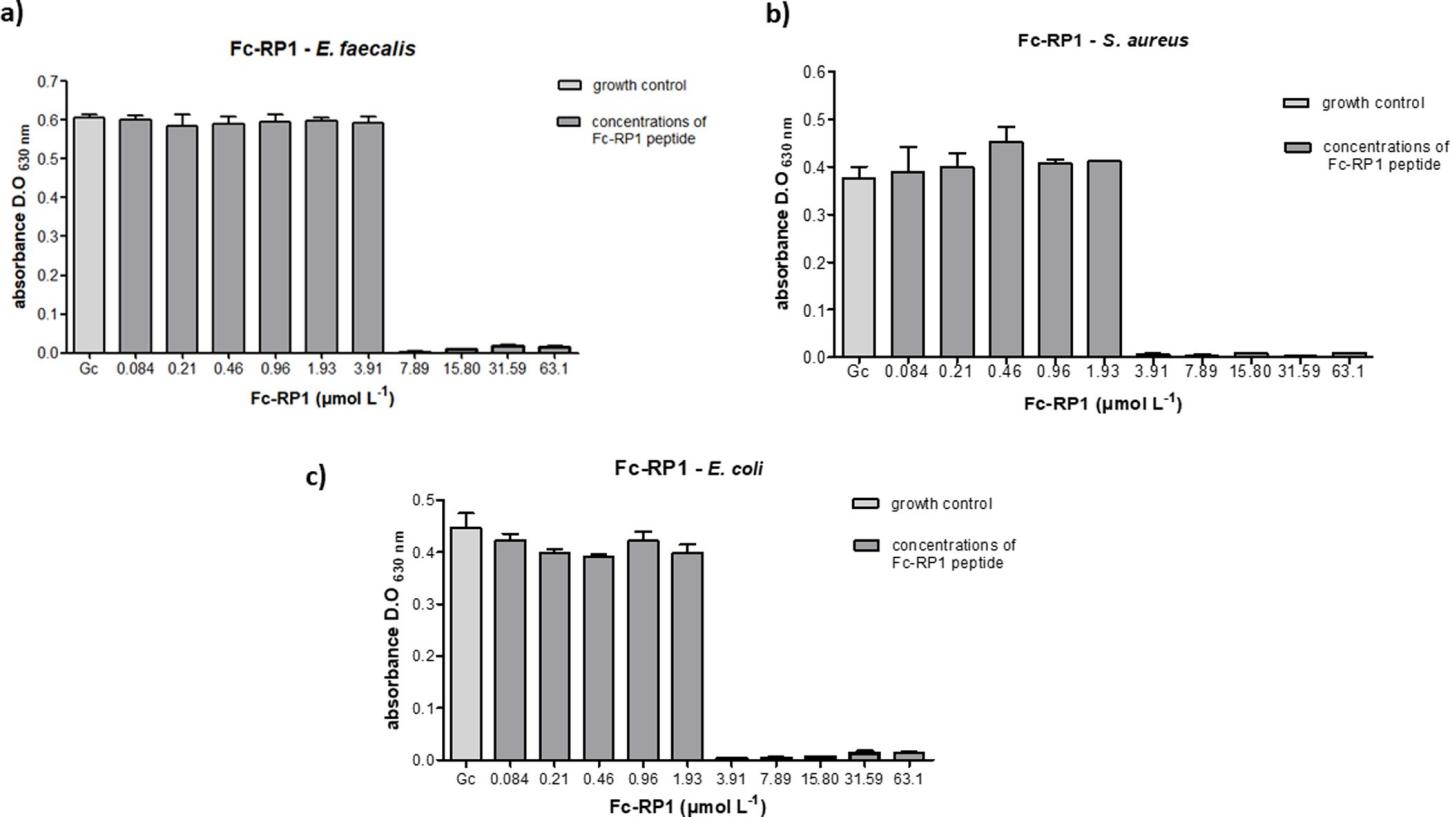
Antibacterial assays with peptide Fc-RP1 against (a) *E*. *faecalis*, (b) *S*. *aureus* and (c) *E*. *coli*. The methodology followed the guidelines of the Clinical and Laboratory Standers Institute (CLSI, 2012). Three independent experiments were performed and the data were analyzed by applying the one-way ANOVA with Tukey's post hoc test using GraphPad Prism Version 5.01 software (GraphPad Software Inc., La Jolla, CA, USA). The maximum coefficient of variation accepted was 25%, the confidence level was 95% (p <0.05) and ***p<0.001, *p<0.05; **p<0.01; ns: not significant.

Regarding the human pathogens, bacteria *S*. *aureus* and *E*. *faecalis* (Gram-positive) and *E*. *coli* (Gram-negative), RP1 peptide was able to completely inhibit the growth of both *E*. *fecalis* and *E*. *coli* (MIC = 8.7 μmol L^-1^). The peptide also showed activity against *S*. *aureus* (MIC = 69.4 μmol L^-1^) (Table 2 and Fig 4), but in higher concentration. As compared with the RP1 peptide, Fc-RP1 showed an improved potency against *E*. *faecalis* (MIC = 7.9 μmol L^-1^), *E*. *coli* (MIC = 3.9 μmol L^-1^) and *S*. *aureus* (MIC = 3.9 μmol L^-1^ and Fig 5) (Table 2), indicating that the synergy of the bioconjugate increased the peptide activity. It is worth to mention that the solution containing only ferrocene carboxylic acid did not cause any effect on bacteria growth (S5 Fig).

**Fig 6:**
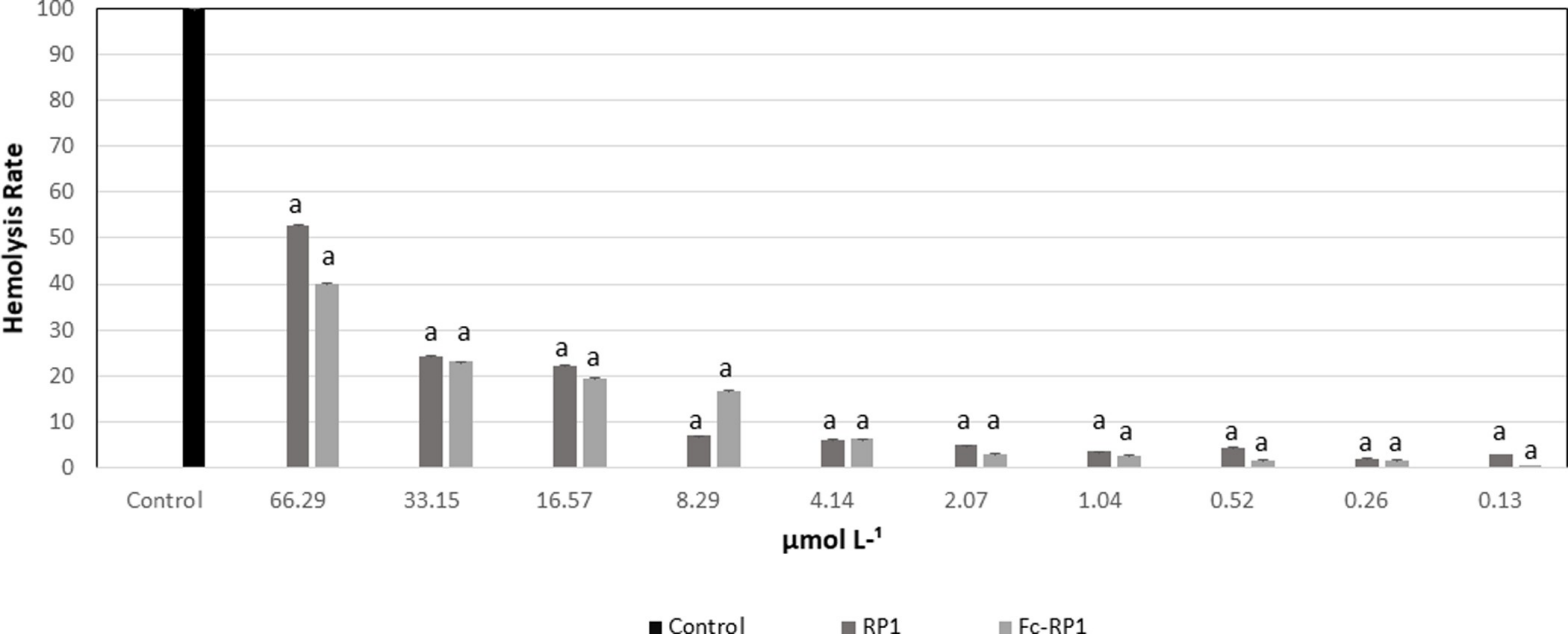
Hemolytic activity of peptides in red blood cells of *Nile tilapia* (*Oreochromis niloticus*). The results are expressed as means (n = 3) with respective standard deviation. Letters differed by Tukey's method (p <0.05).

**Fig 7:**
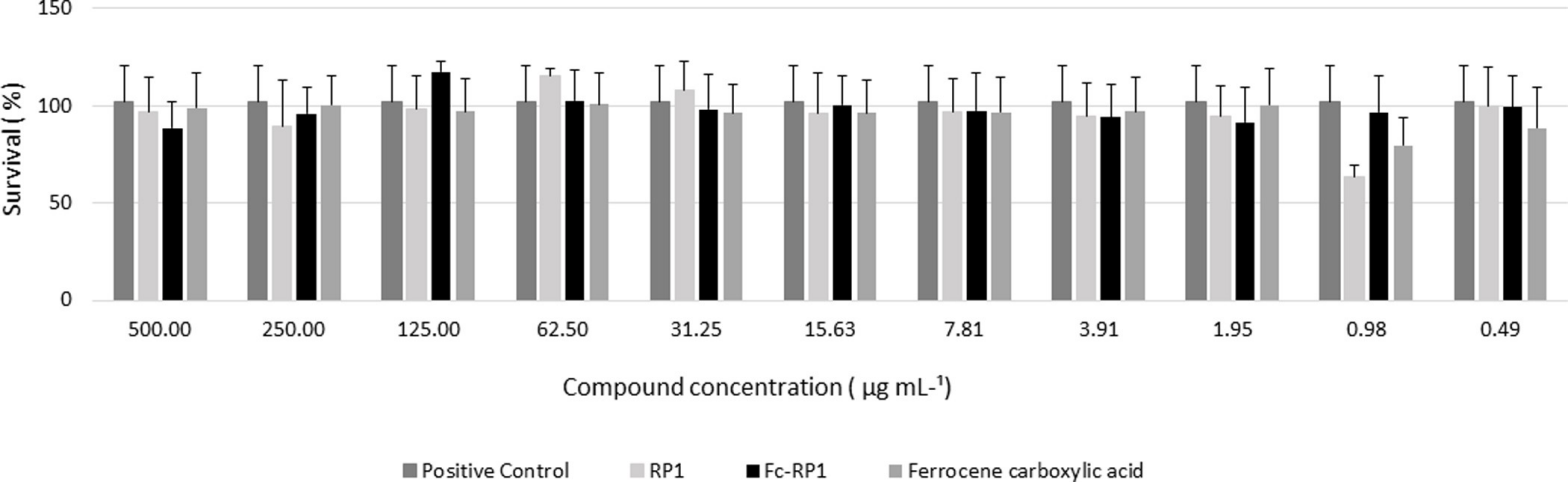
Cytotoxicity tests with the HaCaT (immortalized keratinocyte cell line. All data received statistical treatment (Anova and Tukey's test) and the results presented are not statistically different from each other, the same letter in the Tukey Test.

Moreover, when evaluated the hemolytic activity (HA) of the RP1 and Fc-RP1 on fish RBCs, both compounds caused less than 10% of hemolysis when exposed to a concentration corresponding to the MIC obtained against *S*. *galactiae*, *i*.*e*., 4.30 μmol L^-1^ for RP1 and 0.96 μmol L^-1^ for Fc-RP1. The peptide and the conjugated also did not present any cytotoxicity at the highest concentration tested (220 μmol. L^-1^) (Figs 6 and 7) against HaCaT cells.

### Evaluation of the peptide’s capacity to permeabilize vesicles

**Fig 8:**
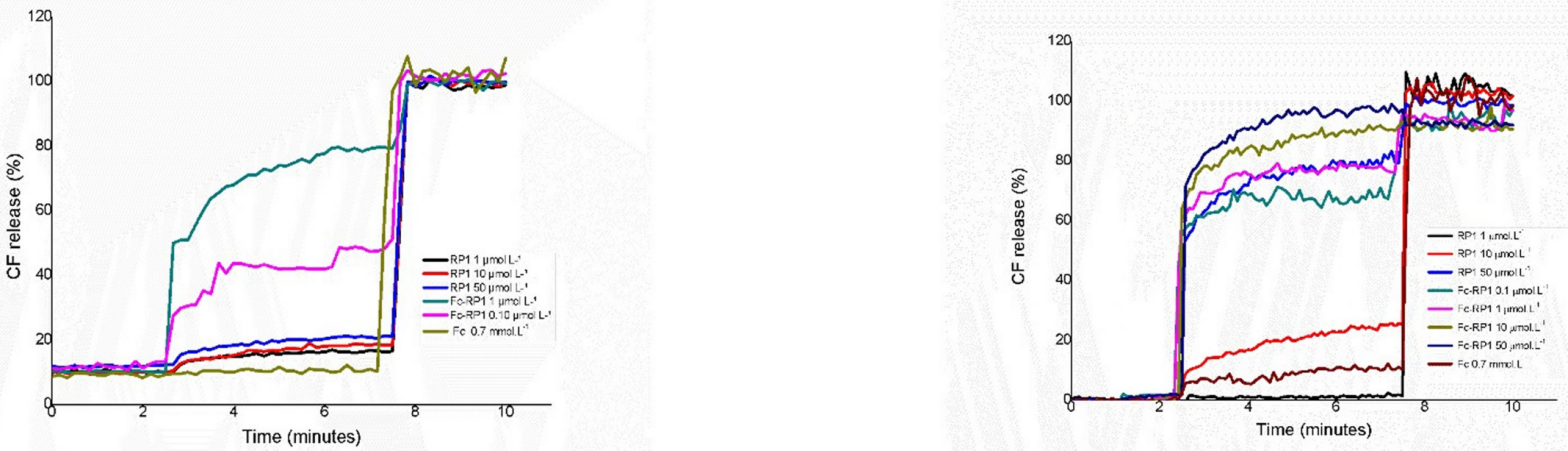
Carboxyfluorescein release by the peptides RP1, Fc-RP1 and the ferrocene carboxylic acid in vesicles of POPC (a) and POPC: POPS (9:1) (b).

In order to understand the mechanism of action of RP1 and Fc-RP1, carboxyfluorescein (CF) release from LUVs were investigated using POPC and POPC: POPS (9:1) vesicles containing this fluorescent marker. RP1 at 50 μmol L^-1^ cause the release of 10% of CF from POPC, while 1 μmol L^-1^ of Fc-RP1was sufficient to release 70% POPC content (Fig 8). Similarly, Fc-RP1 causes higher release of CF (~100%) than RP1 (25%) from POPC/POPS at 10 μmol L^-1^ (Fig 8B), showing that the release of CF is higher in negatively charged phospholipid vesicles than in the neutral ones (Fig 8). This is consistent with the positive net charge of the peptides (+8) in physiological pH. In addition, it is important to highlight that the higher the peptide concentration, the higher the CF release, which indicates the action mechanism by pore formation on the vesicles [63]. The coupling of ferrocene increased the permeabilization ability of the peptide. Several studies have shown that ferrocene destabilize cell membranes through lipid peroxidation, leading to cell lysis [64]. In this study, the ferrocene coupled to the peptide might be improving the lysis of the bacteria membrane, contributing to the observed antibacterial activity. In order to confirm this hypothesis, vesicle permeabilization assays were carried out in the presence of 0.7 mmol L^-1^ of ferrocene carboxylic acid, a compound concentration that is seventy times higher than the previously concentration used when testing the permeabilization potential of Fc-RP1 (Fig 8).

## Discussion

The indiscriminate usage of antibiotics by the population is increasing at the same time as the resistance to medicines, the persistence of infections, and treatment failures. To address this problem, new antibiotic-like molecules that directly kill or inhibit the growth of microorganisms are necessary. In this context, the research about antimicrobial peptides can be of great help. The AMPs have broad activity against different microorganisms, mainly because of their relatively strong electronic interaction with negatively charged membranes [65]. Antimicrobial peptides represent a powerful tool for the development of novel drugs or to complement current therapeutic strategies. Depending on the stage of development of the target parasite, AMPs may have different antiparasitic activity mechanisms [66], for example, by interacting with membrane components, being able to permeate and inducing a variety of cell changes associated with apoptosis [67–69].

Here, the peptide reported, RP1, is derived from the human chemokine CLX4 [36] and modulates immune responses by protecting the host through a series of mechanisms: chemotactic activity, attracting leukocytes; modulation of host cell responsiveness to TLR ligands; stimulation of angiogenesis; increase in activation and differentiation of leukocyte/monocyte; modulation of expression of cytokines/chemokines; and proinflammatory [72–74]. In antiparasitic studies, the peptide can modulate the concentration of IL-12, inducing immune responses, besides stimulating the production of nitric oxide of the host and of IFN λ, making the infected cell to fight the parasite [68, 72, 75–79].

In this work, the ferrocene molecule, one active compound with low levels of in vivo toxicity, was coupled in N-terminus of the peptide RP1 aiming to obtain a more active compound (Fc-RP1). Previous studies showed that compounds conjugated with organometallics, like ferrocene, has great biological potential [38, 40, 52, 70–73].

The RP1 peptide and Fc-RP1 presented anti-amastigote activity in *Leishmania amazonensis* (IC_50_ = 1.25 μmol L^–1^ and IC_50_ = 0.25 μmol L –^1^, respectively). However, the Fc-RP1 conjugated showed higher toxicity (17.3 μmol L –^1^) when in contact with macrophage as compared with the same tests for RP1 peptide and ferrocene carboxylic acid molecule (>100 μmol L^-1^). It is well-known that bioconjugates containing organometallic compounds such as the ferrocene have great biological potential [40, 64, 71, 73]. It is also assumed that this compound acts in the generation of reactive oxygen species. In biological medium, this molecule is oxidized to the cation-radical ferrocene by hydrogen peroxide in the presence of the enzyme peroxidase, inducing cellular stress [37]. The physico-chemical properties, redox process, and the high reactivity on the biological activity of the Fc molecule [74], may explain the increase in the cytotoxic effect of the conjugated [64, 70, 75]. The toxicity of the conjugate can be justified by the lipophilicity property of Fc, that could increase the interaction with the membrane of the macrophage.

Compounds containing Fc have shown anti-proliferative activity on hormone-dependent breast cancer cell line MCF-7 [76], corroborating with our results that showed the Fc-RP1 interacts with macrophage. Moreover, we have demonstrated that N-terminal modifications affect the activity of AMPs [77, 78], with the addition of Fc in the N-terminus changing the peptide charge, which has an important role in driving the membrane selectivity of the AMP peptides. The peptides with acetyl group or Asp residue at the N-terminal region showed activities only against Gram-positive bacteria.

We have also compared the results of the conjugated peptide Fc-RP1 against amphotericin B, which is the second-line drug approved for leishmaniasis treatment. In comparison with amphotericin B (IC_50_ = 0.63 ± 0.72 μmol L-1), the Fc-RP1 was more active, also showing a 2.5-fold higher selectivity index. However, the treatment with amphotericin B presents serious side effects. It is done intravenously and can cause toxicity issues. The cost of treatment using amphotericin B is another obstacle. These problems make that the mortality caused by leishmaniasis continues to rise, requiring more selective and more affordable treatments [79, 80].

In order to evaluate the application of the compounds against bacteria, the antibacterial potential was tested in human pathogenic bacteria: *S*. *aureus*, *E*. *faecalis*, *and E*. *coli*, as well as in some bacteria species that cause disease in fish and humans (*S*. *agalactiae* and *A*. *hydrophila*). The bacteria species *S*. *agalacitiae* and *A*. *hydrophila* are pathogenic for the fish Nile tilapia (*Oreochromis niloticus*), causing a foodborne disease in humans [81, 82]. Often found on the skin and nasal fossae of healthy individuals *S*. *aureus* is a Gram-positive bacterium, considered an opportunistic microorganism and is one of the major causative agents of community and hospital infections worldwide [83] Several diseases are related to this bacterium, from superficial infections of the skin to more serious and deep infections, such as endocarditis, bacteremia, peritonitis, meningitis, necrotizing pneumonia and septic arthritis [84]. Meanwhile, *E*. *coli* is a Gram-negative bacterium found in the intestinal tract, which is also known to cause infectious endocarditis, bacteremia, sepsis and urinary tract infections [83, 85, 86]. Another microorganism of great medical relevance also associated with bacteremias, menigentites, wound infections and urinary tract infections is Gram-positive bacteria *E*. *faecalis*. Additionaly to these diseases, this microorganism is also associated with endodontic infections [87–89].

The antibacterial activity of the peptide in Gram-positive strains can be seen in Fig 5. The RP1 peptide presented a MIC of 4.3 μmol L^-1^ against *S*. *agalactiae*, whilst Fc-RP1 was four times more active (MIC = 0.96 μmol L^-1^), indicating that ferrocene improved the activity against Gram-positive bacteria. The satisfactory activity of the peptide and conjugate on Gram-positive bacteria can be explained by the presence of teichoic acids in their cell membrane, which confers a negative net charge on the bacterial surface, enabling the interaction [90]. Furthermore, the presence of ferrocene could act in the bacterial membrane, interfering in its permeability through pore formation or decreasing the levels of cellular ATP, leading to an energy collapse, and initiating a process of cell death [91]. It is worth mentioning that the ferrocene molecule has already been used in the synthesis of new derivatives of penicillins and cephalosporins [38]. Another fact that could explain the difference between the peptide and the conjugated is the N-terminal modification.

The coupling of ferrocene in RP1 also decreases the minimum inhibitory concentration (MIC) in the assays against *E*. *faecalis* (MIC = 7.9 μmol L^-1^), *E*. *coli* (MIC = 3.9 μmol L^-1^), and *S*. *aureus* (MIC = 3.9 μmol L^-1^), as can be seen in Figs 6 and 7. However, the MIC of RP1 ((MIC = 8.7 μmol L^-1^) and Fc-RP1 (MIC = 7.9 μmol L^-1^) remained similar when tested against *E*. *faecalis* strain, showing that the optimized antimicrobial effect of the Fc-RP1 peptides on the bacteria may be dependent on the evaluated strain. The data also shows that the conjugation of ferrocene to RP1 peptide is an interesting strategy for increasing the bioactivity of the AMP peptides [92–95].

Additionally, the activity of peptides against *E*. *coli* and not against *A*. *hydrophila* could be explained by the interaction of the peptide with other cell wall components. Lorenzon et al. (2013) showed that the dimerized AMP aurein promoted the aggregation of *Candida albicans* via interactions with mannans found in the cell wall. Moreover, the changes in the activity against these strains can be attributed to the capacity of Gram-positive bacteria to modify the composition of their cell membrane [96]. This modification might be done by changes in the concentration of negatively charged phospholipids in the membrane, altering the action of the AMPs.

The cytotoxicity of the compounds was tested against HaCaT cells (Fig 7), helping to evaluate the possibility of the application of these molecules. The molecules did not present any activity at the highest concentration tested (500 μg. mL-1), showing a high potential of using these compounds to develop a new drug.

In order to clarify a possible mechanism of action of the Fc-RP1 peptide, the permeabilization assays were performed. The data shows that the coupling of ferrocene to the RP1 peptide significantly increases the permeabilization of the LUVs. In this study, the ferrocene coupled to the peptide improved the lysis of the bacterial mimetic membrane, in accordance with the antibacterial activity obtained. Besides, several studies have shown that ferrocene destabilizes cell membranes through lipid peroxidation, leading to cell lysis. In order to confirm this hypothesis, vesicle permeabilization assays were carried out in the presence of 0.7 mmol L-1 ferrocene carboxylic acid, which corresponds to a concentration that is seventy times higher than the one used when testing the permeabilization potential of Fc-RP1 (Fig 8). The ferrocene carboxylic acid released 10% of CF, similarly to the data obtained for the RP1, suggesting that the coupling of Fc to the peptide is essential for increasing the permeabilization, probably due to the modification in the charge and the structure of the N-terminal region of the Fc-RP1 [77], leading the peptide to form pores and producing membrane destabilization besides cell damage. Furthermore, the conjugated might be interfering in the membrane potential, resulting in the production of reactive oxygen species and triggering cell death mechanisms [41, 42].

It is noteworthy that even with the Fc-RP1 peptide being a prototype of a bioconjugation strategy; it still is a compound with great biological activity against neglected tropical diseases and fish diseases.

## Conclusion

The solid phase peptide synthesis protocol enabled the development of the conjugate, Fc-RP1, composed of an antimicrobial peptide and the organic molecule ferrocene. The comparison between the AMP and the conjugated Fc-RP1 showed that the presence of the organic molecule increased the antimicrobial activity against *E*. *coli*, *S*. *aureus* and, *E*. *faecalis*, as well as, the activity against a bacterial fish pathogen, S. *agalactiae*. At the same time, the conjugate peptide preserved its low cytotoxicity against HaCaT and fish blood cells. In overall, the obtained data reveals the potential of the Fc-RP1 peptide to be used in antimicrobial therapy, including a possible usage in pisciculture.

## Supporting information

Purity analyzes of the RP1 and Fc-RP1 peptides and their mass spectra are found in the supplemental material (S1 and S2 Figs).

S1 Fig (a) HPLC profile of the crude (a) and purified (b) peptide, with retention time at 10.5 min Analytical HPLC was performed on Shimadzu spectrometer, with C18 reverse phase Ultraspehere Phenomenex column (4.6 mm x 150 mm, 300 Å, particle size 5 μm), detection at 220 nm and gradient method of 5 to 95% solvent B in 30 min with flow rate of 1 mL min^-1^. (c) Peptide mass spectrum profile. The peaks of 721.80; 541.36 and 433.46 represent the mass-to-charge ratio of the peptide RP1 with charge of +3, +4 and +5, respectively.
(TIF)

S2 Fig (a) HPLC profile of the crude Fc-RP1 peptide (Retention time: 14 min) in analytical mode using Shimadzu spectrometer with C18 reverse phase Ultraspehere Phenomenex column (4.6 mm x 150 mm, 300 Å, particle size of 5 μm), detection at 220 nm, using a gradient method of 5 to 95% solvent B in 30 min with 1 mL min^-1^ flow. A peak with retention time at 10.5 min indicates the presence of the uncoupled RP1 peptide. (b) HPLC profile of the pure peptide Fc-RP1. (c) Peptides mass spectrum profile. The peaks of 792.46; 594.61 and 475.86 represent the mass-to-charge ratio of the conjugated with charge of +3, +4 and +5, respectively.
The S3 Fig represents the degradation profile of compounds RP1, Fc-RP1 and Ferrocene Carboxylic Acid at the intervals of 0, 6, 8 and 24 h.
(TIF)

S3 Fig Representation of the degradation profile of compounds RP1, Fc-RP1 and ferrocene carboxylic acid at the intervals of 0, 6, 8 and 24 h. In (a) the compounds were incubated in neutral pH solution (phosphate buffered saline) and in (b) were incubated in acid medium (Water + 0.045% Trifluoroacetic Acid), both images represent the percentage of remaining compounds in different degradation intervals.
S4 Fig shows the activity of peptides RP1 and the conjugate Fc-RP1 against *A*. *hydrophila* (Gram-negative bacteria). It is worth to mention that the ferrocene carboxylic acid did not cause any effect on bacteria growth (S5 Fig).
(TIF)

S4 Fig Antibacterial assays of the peptides RP1 and Fc-RP1 in *Aeromonas hydrophila* (Gram-negative). Peptides diluted in ultrapure water and positive control (bacterium and Mueller Hinton broth). Mean (n = 3) with respective standard deviation. Different letters indicate that the values differ by the Tukey method (p <0.05).
(TIF)

S5 Fig Antibacterial assays with molecule Fc (a) E. faecalis, (b) S. aureus, and (c) E. coli. The methodology followed the guidelines of the Clinical and Laboratory Standers Institute (CLSI, 2012). Three independent experiments were performed, and the data were analyzed by applying the one-way ANOVA with Tukey's post hoc test using GraphPad Prism Version 5.01 software (GraphPad Software Inc., La Jolla, CA, USA). The maximum coefficient of variation accepted was 25%, the confidence level was 95% (p <0.05) and ***p<0.001, *p<0.05; **p<0.01; ns: not significant.
(TIF)
