# Supplementary figures and images for: Antimicrobial activity of RP-1 peptide conjugate with ferrocene group
Source: PLoS One. 2020 Mar 26;15(3):e0228740. doi: 10.1371/journal.pone.0228740 (PMC7098557; doi:10.1371/journal.pone.0228740)

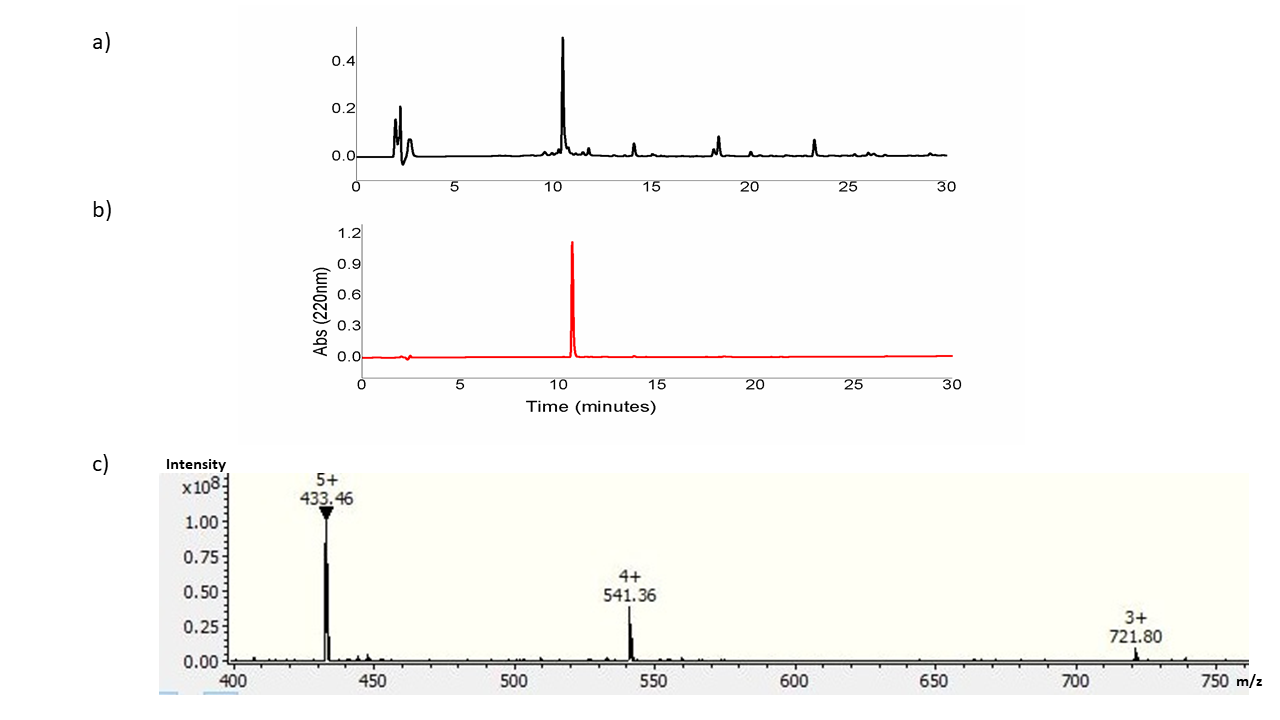

Supplement: S1 Fig — (a) HPLC profile of the crude (a) and purified (b) peptide, with retention time at 10.5 min Analytical HPLC was performed on Shimadzu spectrometer, with C18 reverse phase Ultraspehere Phenomenex column (4.6 mm x 150 mm, 300 Å, particle size 5 μm), detection at 220 nm and gradient method of 5 to 95% solvent B in 30 min with flow rate of 1 mL min-1. (c) Peptide mass spectrum profile. The peaks of 721.80; 541.36 and 433.46 represent the mass-to-charge ratio of the peptide RP1 with charge of +3, +4 and +5, respectively. (TIF) [file pone.0228740.s001.tif]

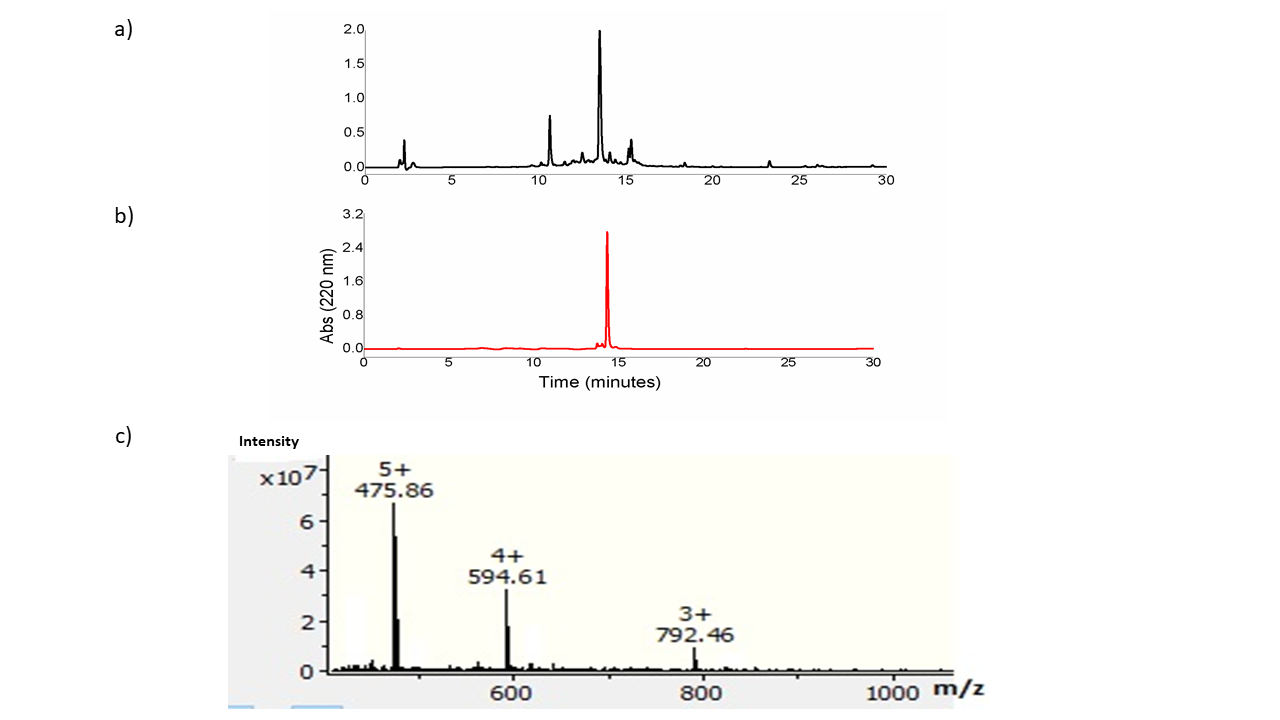

Supplement: S2 Fig — (a) HPLC profile of the crude Fc-RP1 peptide (Retention time: 14 min) in analytical mode using Shimadzu spectrometer with C18 reverse phase Ultraspehere Phenomenex column (4.6 mm x 150 mm, 300 Å, particle size of 5 μm), detection at 220 nm, using a gradient method of 5 to 95% solvent B in 30 min with 1 mL min-1 flow. A peak with retention time at 10.5 min indicates the presence of the uncoupled RP1 peptide. (b) HPLC profile of the pure peptide Fc-RP1. (c) Peptides mass spectrum profile. The peaks of 792.46; 594.61 and 475.86 represent the mass-to-charge ratio of the conjugated with charge of +3, +4 and +5, respectively. The S3 Fig represents the degradation profile of compounds RP1, Fc-RP1 and Ferrocene Carboxylic Acid at the intervals of 0, 6, 8 and 24 h. (TIF) [file pone.0228740.s002.tif]

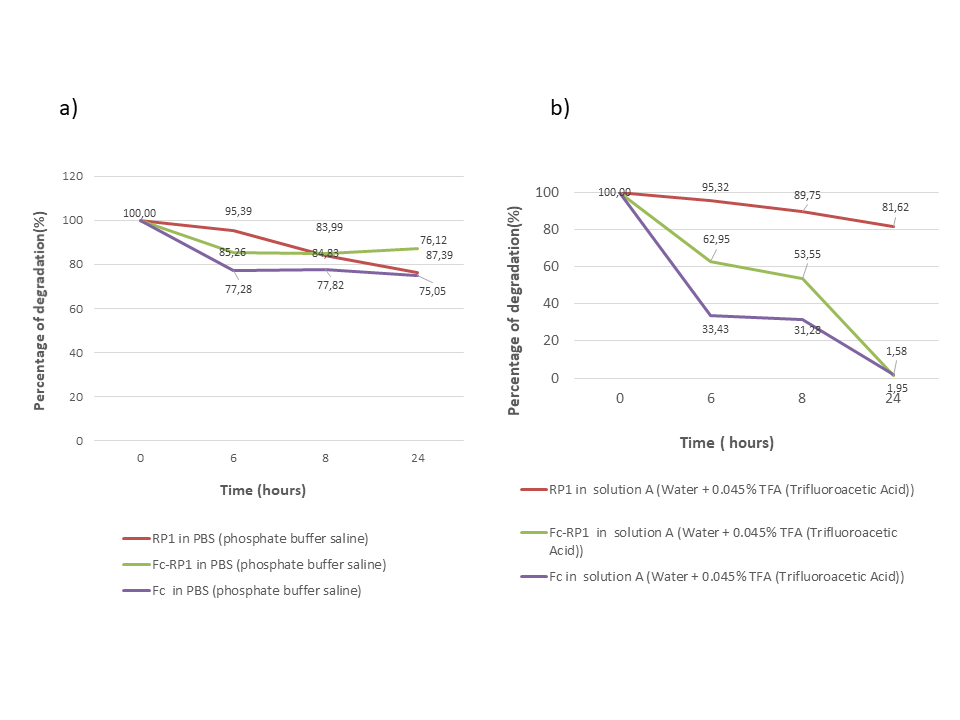

Supplement: S3 Fig — In (a) the compounds were incubated in neutral pH solution (phosphate buffered saline) and in (b) were incubated in acid medium (Water + 0.045% Trifluoroacetic Acid), both images represent the percentage of remaining compounds in different degradation intervals. S4 Fig shows the activity of peptides RP1 and the conjugate Fc-RP1 against A. hydrophila (Gram-negative bacteria). It is worth to mention that the ferrocene carboxylic acid did not cause any effect on bacteria growth (S5 Fig). (TIF) [file pone.0228740.s003.tif]

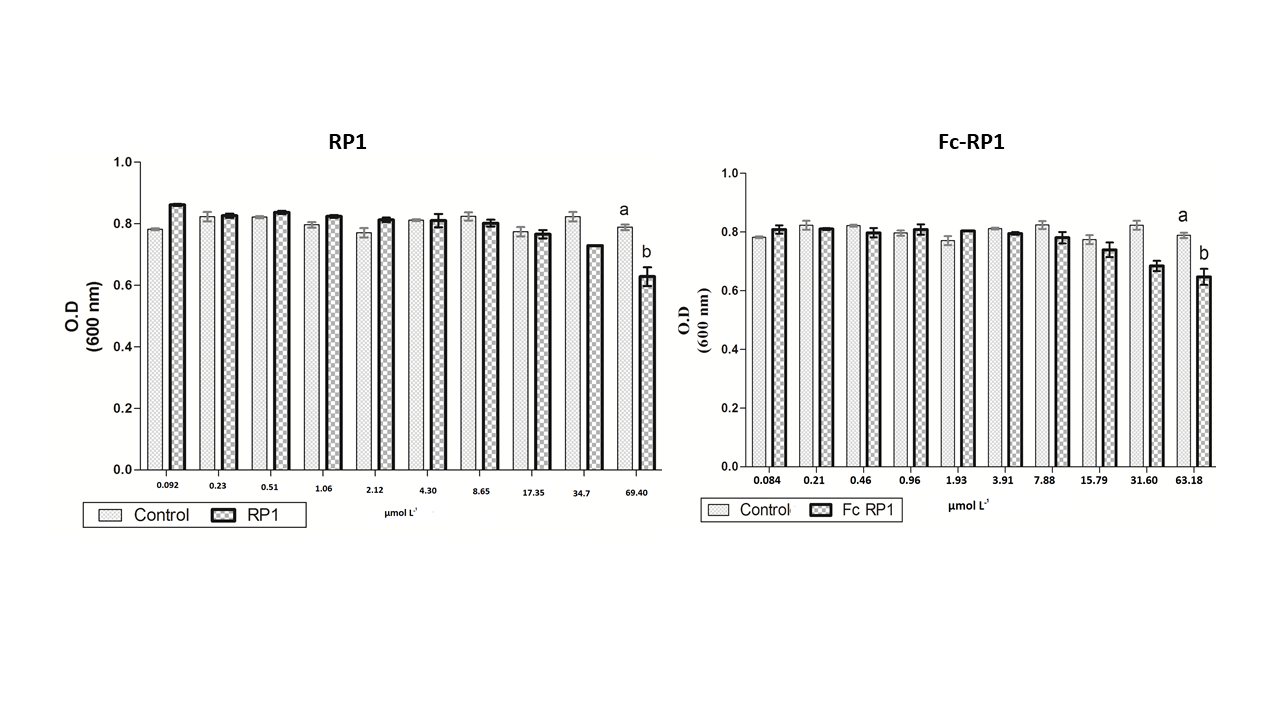

Supplement: S4 Fig — Peptides diluted in ultrapure water and positive control (bacterium and Mueller Hinton broth). Mean (n = 3) with respective standard deviation. Different letters indicate that the values differ by the Tukey method (p <0.05). (TIF) [file pone.0228740.s004.tif]

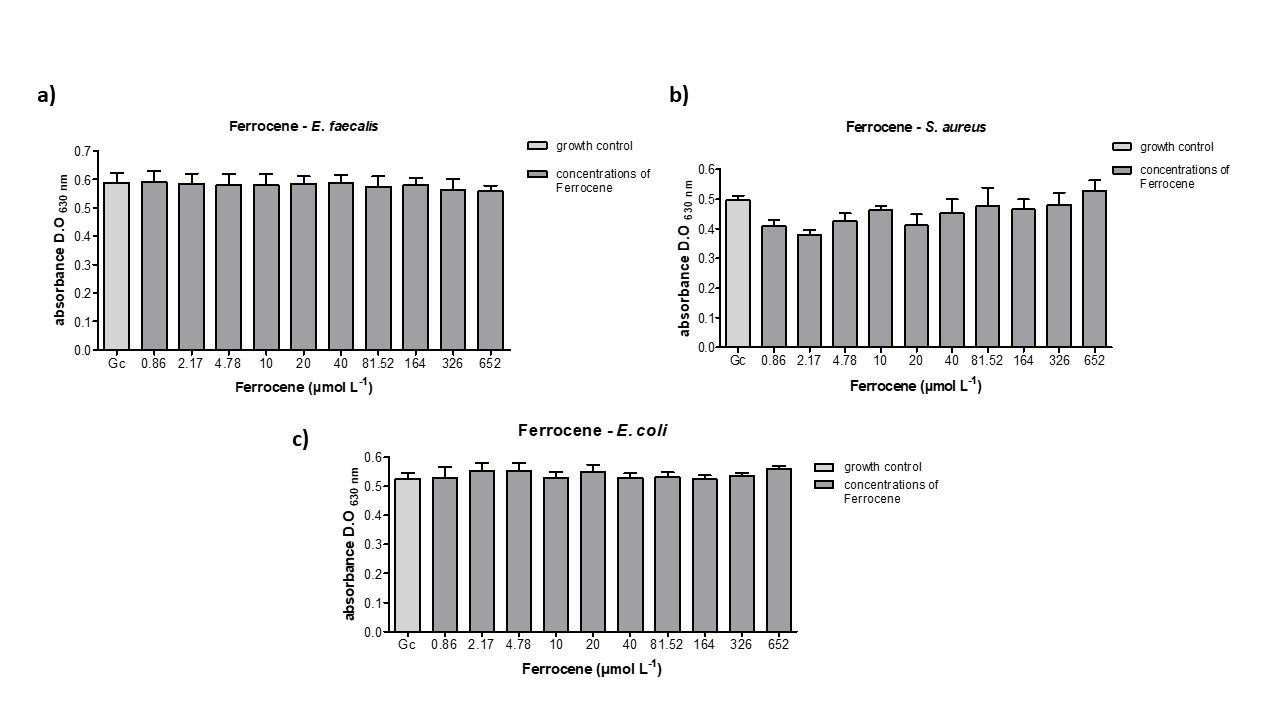

Supplement: S5 Fig — Antibacterial assays with molecule Fc (a) E. faecalis, (b) S. aureus, and (c) E. coli. The methodology followed the guidelines of the Clinical and Laboratory Standers Institute (CLSI, 2012). Three independent experiments were performed, and the data were analyzed by applying the one-way ANOVA with Tukey's post hoc test using GraphPad Prism Version 5.01 software (GraphPad Software Inc., La Jolla, CA, USA). The maximum coefficient of variation accepted was 25%, the confidence level was 95% (p <0.05) and ***p<0.001, *p<0.05; **p<0.01; ns: not significant. (TIF) [file pone.0228740.s005.tif]
